# Bias-Tunable Quantum Well Infrared Photodetector

**DOI:** 10.3390/nano14060548

**Published:** 2024-03-20

**Authors:** Gyana Biswal, Michael Yakimov, Vadim Tokranov, Kimberly Sablon, Sergey Tulyakov, Vladimir Mitin, Serge Oktyabrsky

**Affiliations:** 1 College of Nanotechnology, Science and Engineering, University at Albany, Albany, NY 12203, USA; myakimov@albany.edu (M.Y.); vtokranov@albany.edu (V.T.); soktyabrsky@albany.edu (S.O.); 2 Texas A&M University, College Station, TX 77843, USA; ksablon@gmail.com; 3 School of Engineering and Applied Sciences, University at Buffalo, State University of New York, Buffalo, NY 14260, USA; tulyakov@buffalo.edu

**Keywords:** quantum well infrared photodetector, MWIR-LWIR bands, asymmetrically doped double quantum wells, GaAs/AlGaAs heterostructure

## Abstract

With the rapid advancement of Artificial Intelligence-driven object recognition, the development of cognitive tunable imaging sensors has become a critically important field. In this paper, we demonstrate an infrared (IR) sensor with spectral tunability controlled by the applied bias between the long-wave and mid-wave IR spectral regions. The sensor is a Quantum Well Infrared Photodetector (QWIP) containing asymmetrically doped double QWs where the external electric field alters the electron population in the wells and hence spectral responsivity. The design rules are obtained by calculating the electronic transition energies for symmetric and antisymmetric double-QW states using a Schrödinger–Poisson solver. The sensor is grown and characterized aiming detection in mid-wave (~5 µm) to long-wave IR (~8 µm) spectral ranges. The structure is grown using molecular beam epitaxy (MBE) and contains 25 periods of coupled double GaAs QWs and Al_0.38_Ga_0.62_As barriers. One of the QWs in the pair is modulation-doped to provide asymmetry in potential. The QWIPs are tested with blackbody radiation and FTIR down to 77 K. As a result, the ratio of the responsivities of the two bands at about 5.5 and 8 µm is controlled over an order of magnitude demonstrating tunability between MWIR and LWIR spectral regions. Separate experiments using parameterized image transformations of wideband LWIR imagery are performed to lay the framework for utilizing tunable QWIP sensors in object recognition applications.

## 1. Introduction

Object recognition in the thermal infrared (IR) spectrum can be enhanced by capturing radiation signals in multiple spectral intervals. IR imagers providing on-demand spectral tuning will likely become an essential component of Artificial Intelligence-controlled systems. In particular, the tuning between mid-wave infrared (MWIR, 3–5 µm) and long-wave infrared (LWIR, 8–14 µm) spectral regions provides significant contrast differences making it well-suited for the application of image fusion algorithms [1] in object recognition applications. 

Inter-subband transition-based quantum well infrared photodetector (QWIP) technology based on group III-V heterostructures has attracted much attention over the years for thermal imaging in both MWIR and LWIR spectral regions [2,3,4]. Despite the fast development of other technologies, the high-end applications still value QWIP detectors due to several advantages over the leading competitors, inter-band HgCdTe and GaSb-based superlattice detectors. Some of these advantages are mature large-format GaAs technologies, high uniformity, consistent improvement of performance with temperature reduction for low-background scenes, and high radiation hardness [5,6,7]. Over the last two decades, many theoretical and experimental studies of GaAs/AlGaAs-based QWIPs have been reported targeting the performance improvements and optimization of the QWIP structure and material parameters [8,9,10]. 

A significant breakthrough in thermal imaging was the development of dual-band focal plane arrays (FPAs), in particular operating in both MWIR and LWIR atmospheric windows [11,12,13,14,15], which significantly enhance the visibility of objects and are expected to improve object recognition and identification of object features with different temperatures [16,17]. 

All the dual-band sensor approaches proposed so far utilize separate sensing mediums for two IR bands. In the monolithic sensor designs by Ballet et al. [11] and Smith et al. [12], the inter-band HgCdTe absorbers with different mercury contents and hence different bandgaps were integrated vertically to form two inverted p-n and n-p junctions within a dual-band FPA pixel. This design provides a sequential readout of two spectral images by inverting the pixel voltage bias. Another approach demonstrated by Hu et al. [13] with parallel readout capability used lateral integration of two junctions into two-color pixels with the fill-factor trade-off between the two sensors. Vertically integrated pixels were also demonstrated in the inter-subband QWIP FPAs, typically with independent access to two QWIP sensor arrays [1,14,15]. Each pixel, therefore, had three contact layers, and the FPA provided two separate fixed-spectral-sensitivity images. 

A unique property of the inter-subband transitions explored is that they can be controlled through manipulation of the quantum states by the external bias to tune the QWIP’s spectral sensitivity. Majumder et al. [18] reported a QWIP with voltage tunable detection in a 7–10 μm region. The applied voltage caused the electron transfer between the adjacent QWs with engineered excited states and resulted mostly in a change of the red-tail of the spectral response. Chiang et al. [19] demonstrated voltage-tunable QWIP for LWIR (8–12 μm) detection based on strongly asymmetrical triple-coupled InGaAs/GaAs/AlGaAs QWs. The response spectrum consisted of two major peaks which were blue-shifting with applied bias due to the quantum confined Stark effect. 

Choi et al. [20] recently reported a voltage-tunable infrared detector based on asymmetrically doped double QW for LWIR detection. The tunability was also demonstrated in THz QWIPs [21] and quantum dot IR photodetectors [22]. The bias tunability of QWIP may become a critical property when implemented in an imaging sensor driven by an AI algorithm for autonomous object recognition in the IR [23]. However, so far, no voltage tunability of the sensor between LWIR and MWIR windows has been demonstrated. 

In this work, we present the design and demonstrate a QWIP sensor containing asymmetrically doped double QWs showing voltage-tunable spectral photo-response. The coupled double QW structure was designed to have the absorption in MWIR (~5 µm) to LWIR (~8 µm) regions with the response tunable by the external bias which changes the electron population on the symmetric and anti-symmetric split levels. The sensor was found to have significant sensitivity control making it suitable for recognition of thermal features. To lay a framework for utilizing the tunable QWIP sensor in object recognition applications, we further present separate experiments using parameterized image transformations of wideband LWIR imagery. 

## 2. Materials and Methods

### 2.1. QWIP Design 

**Figure 1:**
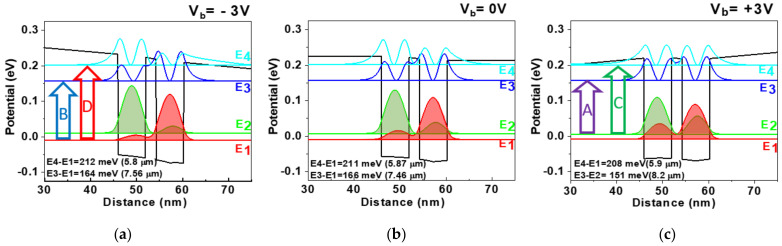
Calculated localized energy states and corresponding wavefunctions (${\left|\psi \right|}^{2}$) in the double QW structure at 77 K under (**a**) −3 V, (**b**) 0 V, and (**c**) +3 V bias. Zero energy corresponds to the Fermi level position. Four transitions are indicated by arrows with the letters “A” to “D” assigned from low to high energies. Structure parameters: QW width, 6 nm; Al content in the barrier, 0.35; barrier width between the coupled wells, 2.5 nm; electron concentration, 5 × 10^11^ cm^−2^.

A finite element one-dimensional Schrödinger–Poisson solver was used to design the asymmetrically doped double QW structures to determine the localized energy states, wave functions, and carrier concentration in the QWs for different biases. The designed conduction band diagram of a single detection unit of the sensor is shown in Figure 1 at three bias voltages, V_b_ applied to 25 QW periods. The unit contains two coupled QWs with one of the QW modulation-doped in the center to provide the potential asymmetry to the structure which helps to improve the sensitivity through re-distribution of the carriers on the split levels with the applied external bias. This redistribution of the electrons occurs due to the Stark effect-related perturbation of the wavefunctions and associated shifts of the corresponding energy levels. A distinctive feature of this structure is the splitting of the ground and excited states into E_1_, E_2_ and E_3_, E_4_ levels, respectively, where the symmetric states (E_1_ and E_3_) are mostly localized in the doped QW and antisymmetric states (E_2_ and E_4_) in the undoped QW. We also note that the splitting of the ground state is typically lesser than that of the excited state due to reduced coupling of the wavefunctions. 

**Figure 2:**
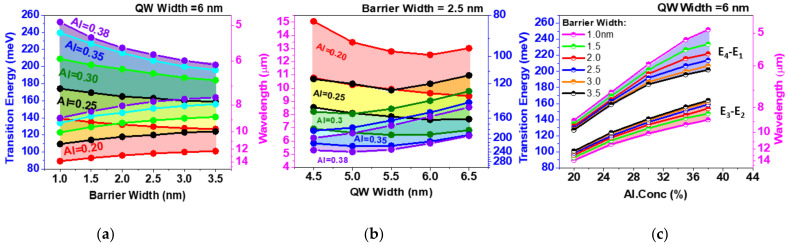
Transition energy and corresponding wavelength (**a**) vs. the internal barrier width in the double QW structure; (**b**) vs. the internal barrier at a constant thickness (2.5 nm); and (**c**) Al concentration in the barrier.

The trends for transition energies and IR detection wavelengths vs. design parameters obtained from the simulation are shown in Figure 2. Figure 2a illustrates the effect of the internal barrier in the double QW structure on the transition energies. The span between the shortest wavelength “D” (E_4_ − E_1_) and the longest wavelength transitions, “A” (E_3_ − E_2_), respectively, corresponds to the range of the QWIP spectral tuning. This range is mostly determined by the coupling of the excited state (E_4_ − E_3_) as long as the E_4_ state stays below the barrier edge keeping the electrons confined. Naturally, stronger coupling through a thinner barrier expands the spectral sensitivity range even touching both LWIR and MWIR regions as in the top left corner of Figure 2a. However, in this case, the responsivity associated with the transitions to the bottom excited E_3_ level may be suppressed as the photoelectrons have to overcome a very high barrier (~100 meV) to contribute to the photocurrent. 

Figure 2b describes the range of spectral sensitivity vs. QW width at a constant thickness of the internal barrier. The transition energies decrease in the pairs of QW wider than 5–6 nm as the excited states (E_4_ and E_3_) are shifting down into the QW. On the contrary, in the narrow QWs < 5 nm, the excited state E_4_ is pushed out from the QWs, and the level splitting (E_4_ − E_3_) is reducing while it is approaching the continuum. 

Aluminum content in the barrier and hence the barrier height is another design parameter of the structure (Figure 2c). An almost linear increase in the transition energies with x in the Al_x_Ga_1−x_As barrier is observed up to the Γ-X crossing of conduction bands at *x*~0.4. Figure 2c also illustrates the spectral range broadening with the barrier thinning, which is the most pronounced in the deep QWs with high *x*. 

QWIP design presented in this paper (Figure 1) targets a wide sensitivity band from LWIR to the MWIR region. On the other hand, the splitting of the excited states was limited to about 50 meV to prevent a drop of the photocurrent from the deeper E_3_ level. The position of the third localized energy level, E_3_, with respect to the barrier conduction band edge is one of the critical parameters that determine the overall QWIP performance. With large E_4_ − E_3_ separation, the escape probabilities from the two excited states to the continuum [4] and respective photocurrents become very different, strongly affecting QWIP tunability. 

The Si δ-doping used in the QW structure corresponds to the optimum doping of 5 × 10^11^ cm^−2^ for maximizing QWIP detectivity at the operating temperature of 77 K [3]. The doping in the center of the right QW of the pair (Figure 1) creates a built-in potential to provide stronger repopulation of the ground state split level with the applied bias. Consequently, the Fermi level placed at zero in Figure 1 is located ~10 meV above the E_1_ filling the first subband but leaving the E_2_ subband relatively empty. 

Figure 1 also illustrates the modification of the wavefunctions as well as the localized energy level with the applied bias, summarized in Table 1. Since only the right QW in the pair is doped, the built-in potential drives electrons mostly into the right QW at zero bias, V_b_ = 0, and just minor redistribution occurs at negative bias, V_b_ = −3 V, on the entire 25-period structure, 89% and 11% in the E_1_ and E_2_ subbands, respectively (Table 1). The positive bias causes a significant redistribution of the electrons in the ground state subbands, 76% and 24% at V_b_ = +3 V. 

### 2.2. QWIP Fabrication

**Figure 3:**
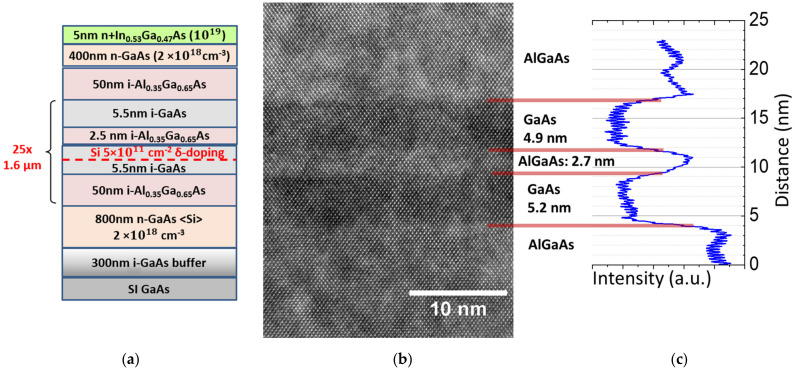
(**a**) Schematic epitaxial structure of the MBE-grown QWIP with asymmetrically doped double QWs. (**b**) High-resolution TEM image of a single detection unit showing GaAs and AlGaAs layers. GaAs appears darker than AlGaAs due to stronger electron scattering. (**c**) Intensity map across the structure to estimate thicknesses.

The QWIP structure was grown using molecular beam epitaxy (MBE) on a semi-insulating 3″ GaAs (001) substrate. The growth sequence is as follows (Figure 3a): starting from an undoped GaAs buffer layer, the detection element containing 25 periods of the double QWs separated with 50 nm thick undoped Al_0.35_Ga_0.65_As barriers was sandwiched between the top and bottom n-GaAs (N_d_ = 2 × 10^18^ cm^−3^) contact layers with thicknesses of 400 nm and 800 nm, respectively. Finally, a 5 nm top In_0.53_Ga_0.47_As heavily doped to a 10^19^ cm^−3^ layer was deposited to improve the contact resistivity. The entire structure was grown at 600 °C except for the top In_0.53_Ga_0.47_As layer grown at 450 °C. Silicon was used for n–type doping of the structure. A cross-sectional high-resolution TEM image of a single detection unit with double QW is shown in Figure 3b along with the corresponding intensity map (Figure 3c) for accurate calibration of the thicknesses to the lattice fringes. It shows reasonable agreement with the theoretical design assuming ~0.5 nm short-range roughness of the interfaces. 

Using standard UV Lithography, wet chemical etching, and metallization techniques, square mesa structures of 200 × 200 µm^2^ and 300 × 300 µm^2^ were fabricated. For wet chemical etching, sulfuric acid (H_2_SO_4_:H_2_O_2_:H_2_O = 1:8:40) was used with an etch rate of 1.7 nm/s. For the top ohmic contacts, Ti/Ni/Ag with thicknesses of 110/130/220 nm, respectively, and for the bottom ohmic contact Pd/Au/Ge/Ni/Ag with thicknesses of 5/30/16/30/150 nm, respectively, were deposited using an e-beam evaporator followed by rapid thermal annealing at 420 °C for 30 s. 

## 3. Results and Discussion

**Figure 4:**
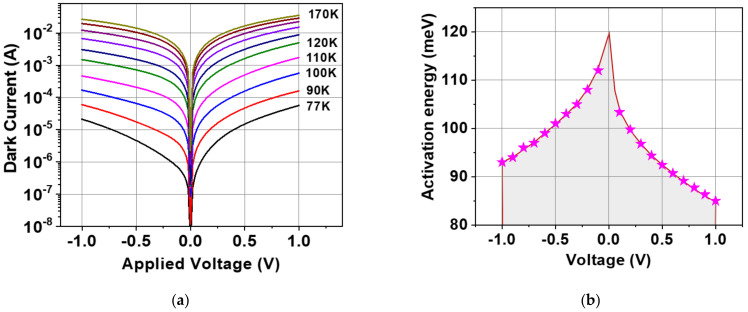
(**a**) Temperature dependence of the dark current in a 300 × 300 µm^2^ QWIP from 77 K to 170 K. (**b**) Activation energy vs. bias from Arrhenius plot for the QWIP.

The variation of experimental dark current with bias at different temperatures starting from room temperature to 77 K is measured using a cryogenic probe station with a warm window with a field-of-view of about 90° and is shown in Figure 4a. Due to the asymmetrical doping in the structure, we observe the asymmetric behavior of the dark current which gradually becomes more symmetric with an increase in the voltage and temperature. At lower temperatures, the density of thermally excited electrons and hence the dark current is reduced by about four orders of magnitude from 170 to 77 K. The curves also indicate that the background-limited temperature (BLIP regime) is below 77 K, which is typical for detectors with LWIR sensitivity [3]. The observed dark current density of ~10^−2^ A/cm^2^ at the applied field of 6 kV/cm and 77 K corroborates the QWs doped to a slightly higher level of about 10^12^ cm^−2^ [3,4]. 

Fitting these temperature dependencies into the Arrhenius equation shows asymmetrical behavior of activation energy with polarity bias, also indicating the significant dependence on the bias. The activation energy of about 110 meV at a low bias (Figure 4b) corresponds to the position of the Fermi level with respect to the barrier. The Fermi level is found to be ~10 mV higher than for the optimally doped single-band QWIP with ~9.5 μm cutoff wavelength [24], which also indicates a slightly higher doping level. The activation energy drops at higher bias likely due to the increased tunneling current from the QWs resulting from image charge barrier lowering [4,24] but can be also contributed by the surface leakage. 

To prove that the proposed design does not degrade QWIP performance, we estimate the major device parameters. The QWIP is mounted inside a continuous LN2 flow cryostat with a KBr window and placed in front of a modulated blackbody source at 700 °C. The photocurrent is measured using a lock-in amplifier. The responsivity of the QWIP is determined from the ratio of the photocurrent to the estimated incident IR power by integration of Planck’s blackbody spectrum at the maximum QWIP spectral peak at 7.5 ± 0.3 μm at −2.5 V bias (see below). This peak corresponds to the maximum responsivity of about *R* = 21 mA/W with possible absolute value inaccuracy of as high as 2–3× due to geometrical and spectral uncertainties. This value of responsivity is used to estimate the integral detectivity ($D*=R\sqrt{A}/i_{GR}$) from the generation–recombination noise current $\left(i_{GR}\right)$ model [24] and is about $2\;\times \;10^{9}\;{cm\;Hz}^{1/2}/W$. The estimated values are reasonably high considering no light coupling elements are used when almost normal-incidence radiation allows for <20% absorption due to polarization selection rules [4,25]. 

**Figure 5:**
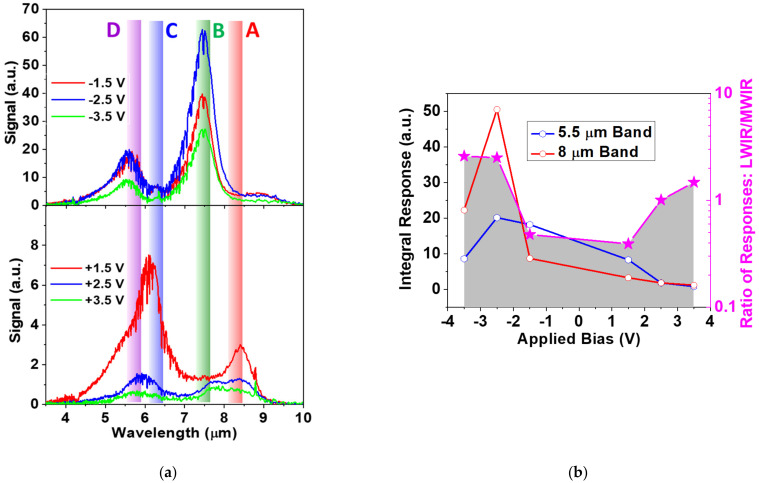
(**a**) Experimental FTIR spectral response of 25-period QWIP at 77 K as a function of applied bias for positive and negative polarities compared with transition energies obtained from the simulation in Figure 1. The transitions are indicated by wide lines extended to higher energies qualitatively resembling the spreading of the electrons in the respective subbands. Transistions “A” to “D” are indicated in Figure 1. (**b**) Integral response of the two bands controlled by the applied bias to the QWIP structure and the response ratio of the two bands at 5.5 µm and 8 µm.

Spectral photo-response of the detector is measured using a Fourier transform infrared (FTIR) spectrometer (Figure 5a). The cryostat with the detector is placed outside the N_2_-purged spectrometer enclosure; therefore, multiple H_2_O and CO_2_ absorption lines are present in the spectra. The detector response reveals the presence of four detection bands corresponding to transitions “A” to “D” labeled in Figure 1. 

We observe a well-pronounced spectral sensitivity control from a 7.5–8 µm peak for the negative bias (Figure 5a) to a 5.5~6 µm peak for the positive bias (Figure 5a). Under the negative bias, the electric field in the QW pairs adds up to the built-in field due to modulation doping in the right QW and further pushes the electrons from the undoped QW to the doped QW. As a result, “B” and “D” transitions from the E_1_ subband dominate in the spectrum (Figure 1a).

The positive bias reduces the built-in field in the QW pairs pushing electrons back into undoped QW thus populating the E_2_ subband (Table 1). This leads to the appearance of transitions “A” and “C” from the E_2_ subband in the spectrum. The repopulation of electrons in the split level and coupling in the excited energy levels with the continuum is mostly responsible for the spectral tuning observed in the FTIR spectrum for different voltages. Increasing the bias in both positive and negative polarity reduces the lifetime of the electrons on the excited states against tunneling into a continuum resulting in a drop in response. This effect is more pronounced for the top E_4_ level; at positive bias, response “C” starts decreasing at <−1.5 V (Figure 5a), but the strongest transitions to the E_3_ level at the positive bias, “B”, drop at >+2.5 V. 

To quantify the voltage tunability observed for the sensor, the integral responses of two major detection bands (LWIR “A + B” and MWIR “C + D”) are plotted in Figure 5b. The ratio of the LWIR-to-MWIR integral responses shows a significant bias control of the responsivity of about an order of magnitude, making the sensor a promising candidate for the detection in MWIR to the LWIR region. The sensitivity control can likely be further improved by optimization the splitting of the excited state in the double QW structure which controls the trade-off between the spectral response range and QWIP responsivity at the long wavelengths associated with the E_3_ level (peaks “A” and “B”).

The voltage-tunable spectral response property of the QWIP can help in multiwavelength IR detection in the MWIR-to-LWIR range. Voltage tunability enhances the sensor’s adaptability to the environment by allowing for the adjustments to the sensor’s spectral response and provides on-demand sensitivity adjustments by the Artificial Intelligence-driven object recognition algorithms. The fabrication of an adaptable focal plane array using the proposed technology will likely improve AI-based autonomous object recognition applications in the thermal infrared spectra.

## 4. Object Detection for Parametrized Spectrum Images

The spectral response of the developed QWIP sensor changes with the applied bias voltage. Thus, the sensor is capable of collecting multiwavelength or multichannel IR image data on demand similar to RGB channels captured by cameras operating in the visible light spectrum. Our recent work in Ref. [23] showed that such multiwavelength IR images can result in higher object detection rates than the images consisting of a single wide band IR channel. While in [23], the authors experimented with a fixed narrow band of the IR spectrum obtained with the help of an additional IR filter and uncooled camera, the developed cooled sensor is actually capable of capturing a variable range of IR spectral bands defined by a voltage parameter with higher signal-to-noise ratio. Continuing our efforts in [23], we constructed a framework for object detection and fusion of the detection results which can be applied to such spectral band channels of the developed QWIP sensor. Since it is not yet possible to obtain the images with the proposed multiwavelength channel characteristics, we developed the framework using the parameterized IR image transformations, where the change in parameter is analogous to the change in the base voltage of the QWIP sensor.

We conducted a series of experiments using the data collected by a commercially available LWIR camera—FLIR Boson 640. The raw images collected by the sensor consist of single-channel 16-bit IR intensity data. The existing object detection algorithms typically work on one- or three-channel 8-bit images, and to perform the object detection in the camera’s images, 16-bit images need to be converted to 8-bit images. To simulate the workings of the developed QWIP sensor, we created such parameterized transformation where the change in parameter mimics the change in the base voltage in the sensor. 

As a base for our construction, we considered a histogram equalization image transformation. It can be represented as a two-stage function $HE\left(x\right)=M(D\left(x\right))$ where D is a cumulative distribution function of the intensity values of the pixels in a given image with the range of [0, 1] and M is a discretized mapping to the output pixel values, {0, …, 255}. Then, the constructed parameterized transformation can be represented as $F_{p}\left(x\right)=M(S_{p}(D\left(x\right)))$, where $S_{p}$ is a variation of a superellipse function: $S_{p}\left(y\right)={\left(1-{\left(1-y\right)}^{p}\right)}^{1/p}$. Note that $S_{p}$ is a mapping from [0, 1] to [0, 1], $S_{p}\left(0\right)=0$, $S_{p}\left(1\right)=1$, $S_{1}\left(y\right)=y$, and for considered values of p<1, $S_{p}$ is a concave-up function with slopes less than one near zero and bigger than one near one. Thus, the whiter pixels corresponding to the values of $D\left(x\right)$ near one are mapped to larger interval after $S_{p}$ than darker pixels; they are assigned a larger range of output pixel values and as a result receive more emphasis after transformation.

In the experiments, we addressed the problem of car detection in adverse weather conditions. The experimental data were collected at dusk and night on the public highways during winter storm with sleet and snow conditions. The images were captured at discrete time intervals while triggered by a motion detection algorithm. The collected data consist of 1286 images with 4073 manually labeled car objects.

**Figure 6:**
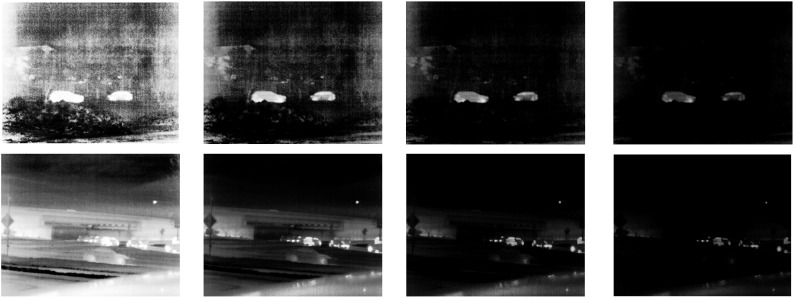
Examples of 8-bit images obtained by parameterized transformations from raw 16-bit images.

Figure 6 shows two examples of collected images with each raw 16-bit image being transformed into g=four different 8-bit images for parameter values $p=1,\;\frac{1}{2},\;\frac{1}{3},\;\frac{1}{4}$. The first parameter value, p=1, actually results in the baseline histogram equalization image processing, and for other values of p, we obtain the images that increasingly emphasize the regions with higher pixel intensity values. As these images illustrate, different transformations can result in better visibility of different car objects; the cars that are well distinguished in one transformed image might be indistinguishable in others due to either oversaturation or undersaturation of the image region containing the object. 

We utilized the YOLO v4 deep convolutional neural network [26] pretrained on the MSCOCO dataset as our base object detector. This network seems to perform well on the LWIR images even without fine-tuning [27]. The results of object detection (only car class results are considered) are presented in Table 2. The numbers are the mean average precision (mAP) in % averaged over different detection IoU (Intersection over Union) thresholds: mAP @ IoU = [0.50:0.05:0.95]. The parameterized transformations for all considered values of p<1 show significantly better performance than the baseline histogram equalization transformation corresponding to p=1. 

Although it is possible to find a particular parameter value that maximizes the object detection performance and use this value in future applications, we hypothesized that better performance can be achieved by the fusion of the detection results obtained with different values of the parameter. In particular, we considered the fusion of detection results on images transformed by histogram equalization and our constructed parameterized transforms. We considered a simple confidence score-based fusion algorithm in the experiments: if two detection candidates from two images have similar bounding boxes, then we take an average of box positions and average score as a fused candidate; otherwise, in case of no match, we reduce the confidence of the non-paired candidate by a fixed constant. The results of the fusion experiments are presented in Table 3. In all cases, the fusion improves upon the performance of any single detection used in the fusion. 

The presented algorithm has related research in the field of photography and image processing algorithms for the traditional visible light spectrum. Our constructed parameterized transforms can be compared to the variation of the exposure time in traditional cameras. While many algorithms for automatically detecting exposure time or other camera parameters rely on some heuristically constructed image quality measures [28], some newer works try to derive the camera parameters based on object detection performance [29]. From another point of view, the raw images from the used LWIR camera can be seen as analogous to the images produced by HDR cameras. By changing the parameters of the HDR image processing algorithms, it is possible to extract more confident features from very dark or very bright image regions, which can benefit subsequent computer vision performance [30]. 

While we achieved positive results with parameterized transformations and the fusion of object detection results from obtained images, the images produced by the developed QWIP sensor have a different nature. In the performed experiments, we observed a direct relationship between parameter p and pixel intensities of transformed images. For the QWIP sensor, the change in base voltage has a more complex relationship with the captured images due to the nonlinear emission spectrum of objects being imaged, as well as the complex form of sensor response spectra emphasizing either MWIR 5.5~6 µm or LWIR 7.5–8 µm regions (Figure 5a). Nevertheless, we expect the presented object detection network will be successfully ported to the new imagery. 

The distinguishing feature of our presented work is the use of multiple image transformations and the fusion of the detection results. This stands in contrast to existing works like [28,29] searching for single best image control parameters. Effectively, such existing works are equivalent to the attempt to find the best color channel to recognize the objects. But, by using fusion, we essentially try to fuse the multiple color channels, e.g., related to 5.5~6 µm and 7.5–8 µm wavelengths, to obtain the best object detection performance, which should lead to superior results. Clearly, the presented choice of parameterized image transformations and the fusion algorithm is rather simple, and multiple improvements, e.g., automated, or machine learnable, choice of transformation functions, better fusion algorithm, etc., can be developed for it and will be a focus of our future work.

Note that the developed algorithm to enhance object recognition with the help of parameterized image transformations can be readily applied to the 16-bit images captured by existing commercial LWIR cameras. This application is optional—it works on captured images and does not impact the camera workflow. For the tunable QWIP sensor, though, the adaptable voltage parameterization has a more important role. For example, imaging of a moving object limits the number of captured frames, and the sensor has to choose the bias parameter which optimizes the object recognition results. Thus, object recognition and fusion could be important components of the sensor. 

## 5. Conclusions

We fabricated, characterized, and analyzed a voltage-tunable MWIR/LWIR sensor based on the asymmetrically doped double QW structure. The two major absorption peaks of our device were observed at about 5.5 to 8 µm with changing applied bias from −4 V to +4 V. The demonstrated voltage tunability is the result of the interaction of multiple processes occurring in the QWIP: Stark effect shifts of the localized energy levels in the QWs and the alteration of electron populations on the split levels/subbands with the applied electric field. Voltage tunability for the sensor is realized by the ratio of integral response of the two detection bands showing an order-of-magnitude control of sensitivity with an applied bias of +/−4 V. Within a feasible framework of utilizing a tunable QWIP sensor, experiments with commercial LWIR imagers included parameterized image transformations and the fusion algorithm to improve object recognition applications.

## Figures and Tables

**Table 1:** Calculated electronic transition energy and relative electron population on the split ground-state subbands in the double QW structure from Figure 1.

	E_1_→E_4_ (D)	E_1_→E_3_ (B)	E_2_→E_4_ (C)	E_2_→E_3_ (A)
**−3 V**	212 meV (5.8 µm)	164 meV (7.6 µm)	192 meV (6.4 µm)	147 meV (8.4 µm)
**Relative population**	89%		11%	
**+3 V**	208 meV (5.9 µm)	164 meV (7.6 µm)	195 meV (6.3 µm)	151 meV (8.2 µm)
**Relative population**	76%		24%	

**Table 2:** Object detection performance on parameterized images.

*p* = 1 (HE)	31.31
*p* = 1/2	40.54
*p* = 1/3	**41.06**
*p* = 1/4	35.27

**Table 3:** The fusion of detection results.

	Fusion
*p* = 1 & *p* = 1/2	40.59
*p* = 1 & *p* = 1/3	**41.96**
*p* = 1 & *p* = 1/4	39.32

## Data Availability

The data presented in this study are available on request from the corresponding authors.
